# Testimonial to Dr. Conolly

**Published:** 1850-10-01

**Authors:** 


### TESTIMONIAL TO DR. CONOLLY.

A NUMBER of professional friends and admirers of Dr. Conolly have originated a subscription, with the view of presenting to this physician a public testimonial, indicative of the respect entertained towards him for his exertions in ameliorating the condition of the insane confined in the institution at Hanwell, over which he has for so many years presided. We need not say that the proposition meets with our warmest approval.

